# Supplementary material for: The role of SNAP and WIC participation and racialized legal status in U.S. farmworker health
Source: PLoS One. 2022 Aug 17;17(8):e0272911. doi: 10.1371/journal.pone.0272911 (PMC9385050; doi:10.1371/journal.pone.0272911)
Supplement: Attachment — Submitted filename: Response to Reviewers.docx [file pone.0272911.s001.docx]

June 29th, 2022

Dr. Alejandra Clark

Division Editor

PLOS ONE

**MANUSCRIPT REVISION**

Dear Dr. Clark,

Enclosed, please find the revised manuscript (Manuscript Reference Number: PONE-D-21-36136), “The role of SNAP and WIC participation and racialized legal status in U.S. farmworker health.” We agree with the reviewer that this manuscript addresses an issue of great importance as it highlights the significant racial and ethnic differences in self-reported health among a primarily Latinx workforce. In addition, we highlight the health-protective potential offered by public assistance programs like SNAP and WIC, particularly for farmworkers who may be less likely to participate.

We are pleased to submit a revised version of the manuscript that addresses the points raised during the review process. We appreciate the comments from the editor and reviewer and have provided specific responses to the comments in the table that follows*.*

We are grateful to submit this revised manuscript. We have attached two versions of the manuscript: one version with tracked changes to demonstrate the revisions, and a clean version without markup. Please let us know if you have any questions or need additional information.

Sincerely,

Briana Rockler, Ph.D. M.P.H.

Public Health and Environmental Studies

University of Wisconsin – Eau Claire

364 Hibbard Humanities Hall	| 124 Garfield Ave

Eau Claire, WI 54702

[rocklebe@uwec.edu](mailto:rocklebe@uwec.edu)

| **Comments to Authors** | **Authors’ Response** |
| --- | --- |
| Journal Requirements | |
| Please ensure that your manuscript meets PLOS ONE's style requirements, including those for file naming. | We have checked to ensure the manuscript meets PLOS ONE’s style requirements, including those for file naming. |
| Your ethics statement should only appear in the Methods section of your manuscript. If your ethics statement is written in any section besides the Methods, please delete it from any other section. | Ethics statement appears at the end of the Methods section of the manuscript (lines 192-193). |
| Please review your reference list to ensure that it is complete and correct. | The reference list has been edited to ensure it follows the ICMJE style guidelines and to rearrange some references in accordance to the changes detailed below (lines 412-534). None of the cited articles have been retracted. |
| Reviewer’s Comments | |
| For clarity purposes please ensure that any acronyms (e.g. SNAP) are spelt out in the abstract. | We clarified the acronyms SNAP and WIC in the abstract (lines 20-22). |
| To ensure that your manuscript meets our fourth publication criterion please address the reviewer's recommendation with respect to the presentation of your results and references to statistical significance. | Thank you for this comment. The review prompted us to re-analyze the data and make several edits that further strengthened the manuscript. In consideration of the reviewer’s recommendations regarding the presentation of results and references to statistical significance, we made the changes described below to address concerns 1-3. |
| 1. One minor area of concern is that the authors use the term "approached significance" or near-significant (p=0.07) describing the relationship between self-reported health status and legal immigration status. It would be more accurate to say simply that this relationship was not significant. Because of the multiple variables considered in table 1, one could argue that even p< 0.05 is a generous threshold for significance, given the problem of multiple comparisons. | Upon re-analyzing data to address the reviewer’s recommendation, legal status was found to be a significant variable in chi-square tests of independence (Table 1). Analyses using linear regression remained not significant and thus, the original conclusions from this study were upheld. In considerations of the reanalysis and the reviewer’s recommendation, we have made the following changes to clarify the relationship between self-reported health (SRH) and legal immigration status:   (1) In the abstract (lines 31, 36-37) we clarified that farmworkers reporting excellent or good health were more likely to be U.S. citizens, but there was no significant association between SRH and legal status (2) In the results we reported significant differences in SRH by legal status (Table 1 and line 217), but no association between SRH and legal status (Table 2 and lines 232-235), except for unauthorized immigrant farmworkers who participated in SNAP or WIC (table 3 and lines 257-261). (3) In the discussion we state that there was no overall association found between SRH and legal status (lines 291). Further, we draw upon the published literature to contemplate the relationship between SRH and legal status (lines 295-316). (4) In the conclusion section, we reiterate that legal status has no direct association with farmworker SRH (lines 400-401). |
| 2. I also found the tables to be somewhat difficult to interpret. Fair and poor were collapsed due to sample size issues, but I could see collapsing Excellent and Good into one category (dichotomizing the SRH into excellent / good vs fair/poor) would make sense and be more interpretable. | This was an important comment. We believe that by re-analyzing the data after collapsing both Excellent and Good into one category we have improved the interpretability of the tables. Tables and figure have been updated for interpretability and to reflect reanalysis (lines 204-209, 236-241, 262-264, 266-272). |
| 3. The sentence (lines 276-280) in the discussion where the authors wrestle with the lack of association between SRH and legal status is confusing and perhaps self-contradictory, in part because the authors seem committed to making the case that the study results confirm their conceptual framework of racialized legal status as a predictor of health outcomes. In contrast, the sentence (lines 283-284) is quite clear, succinct, and represents the actual findings that a) there are significant racial-ethnic differences in SRH b) there are no significant differences in SRH by legal status c) participation in SNAP/WIC was associated with significantly better SRH in Hispanic farmworkers. | We agree that this sentence was unclear. We removed the sentence (lines 321-324) and reorganized the discussion section to clarify the findings and conclusions of this study (lines 285-394). We were especially careful to focus upon the points highlighted by the reviewer with the following revisions:  “This study provided two main findings regarding RLS as a social determinant of farmworker health. First, results confirmed disparities in farmworker SRH by race/ethnicity but not by legal status. Second, participation in SNAP or /WIC participation may modify the negative association between SRH and race/ethnicity.” (lines 285-288)  “These findings suggest that race and ethnicity may be more salient constructs associated with excellent and good SRH ratings among farmworkers.” (lines 292-293)  “We found that the Hispanic farmworkers who indicated participation in SNAP or WIC were more likely to report excellent or good SRH than non-Hispanic White farmworkers using the same programs.” (lines 328-330)  “Participation in SNAP and WIC may be pathways through which RLS determines Hispanic farmworkers’ health.” (lines 347-348) |
